# Effect of Cooling Rate on the Microstructure and Mechanical Property of Nickel-Based Superalloy MAR-M247

**DOI:** 10.3390/ma17050982

**Published:** 2024-02-20

**Authors:** Yue Wang, Jinshan He, Pinpin Hu, Chengbo Xiao, Xitao Wang

**Affiliations:** 1 Collaborative Innovation Center of Steel Technology, University of Science and Technology Beijing, Beijing 100083, China; wangyue595@126.com; 2 Science and Technology on Advanced High Temperature Structural Materials Laboratory, AECC Beijing Institute of Aeronautical Materials, Beijing 100095, China; pinpin.hu@biam.ac.cn (P.H.); cbxiao0288@sina.com (C.X.); 3 Shandong Provincial Key Laboratory for High Strength Lightweight Metallic Materials, Advanced Materials Institute, Qilu University of Technology (Shandong Academy of Science), Jinan 250353, China

**Keywords:** MAR-M247 superalloy, cooling rate, microstructure, tensile property, deformation mechanism

## Abstract

Heat treatment is an important process for optimizing the microstructures of superalloys, and the cooling rate after solid solution treatment is one of the most critical parameters. In this work, we treated solid solution MAR-M247 alloys with water quenching, air cooling, and furnace cooling. Microstructure characterization, hardness, and room temperature tensile tests were conducted to investigate the effect of cooling rate on the microstructure and mechanical properties of MAR-M247 alloys. The results showed that the cooling rate after solid solution treatment mainly affected the precipitation behavior of the secondary γ′ phase, but it had few effects on other microstructure characterizations, including grain size, γ/γ′ eutectic, and MC carbide. The water-quenched sample had the highest cooling rate (400 °C/s) and hardness (400 HV) but suffered from premature fracture because of quenching cracks. A further decrease in cooling rate from 1.5 °C/s to 0.1 °C/s deteriorated hardness (384 HV to 364 HV) and yield strength (960 MPa to 771 MPa) but increased elongation (8.5% to 13.5%). Moreover, the deformation mechanism was transformed from dislocation shearing to Orowan bypassing. The decreased yield strength was mainly due to the weakened precipitation strengthening resulting from γ′-phase coarsening. The improved elongation was attributed to not only the higher work-hardening index caused by interface dislocation networks but also the more uniform deformation, which delayed necking.

## 1. Introduction

MAR-M247 is a polycrystalline nickel-based superalloy and is widely employed in the aerospace industry in the production of advanced turbine blades and rotating parts due to its excellent casting properties and mechanical strength [1,2,3,4]. In order to improve its mechanical properties, many elements have been added to MAR-M247. Elements such as Cr, W, and Mo can dissolve in the γ matrix to produce solid solution strengthening [5,6]. Al and Ti primarily contribute to precipitation strengthening in the form of γ′ [7,8,9]. B and C prefer to form compounds to pin grain boundaries [10,11,12]. However, the high percentage of refractory elements (Ta + W + Mo) causes elemental segregation during solidification and the formation of the γ/γ′ eutectic in the interdendritic regions [13,14]. Therefore, solid solution and aging treatments are usually applied to further improve mechanical properties [15]. Heat treatment mitigates elemental segregation, refines the dispersion of γ′ and carbides in the γ matrix, decreases the amount of the γ/γ′ eutectic, and optimizes carbide morphology and distribution at the grain boundary [16].

In recent years, efforts have been devoted to the study of the heat treatment of MAR-M247. Wolff et al. [17] performed a solution heat treatment in multiple steps, increasing the temperature to 1230 °C for 2 h and then to 1260 °C for 2 h. The multiple-step treatment increased the incipient melting point of the alloy, and more than 90% of the γ/γ′ eutectic had dissolved. Lee et al. [18] found that after being solution-treated at 1240 °C for 2 h, primary γ′ could decompose completely into secondary γ′. Li et al. [19] studied the influence of solid solution temperature on the microstructure and the stress rupture properties of MAR-M247 alloys. Their results showed that, with the solid solution temperature increasing from 1175 °C to 1230 °C, the amount of the γ/γ′ eutectic phase decreased, and the amount of the fine regular γ′ phase increased. With increasing solid solution temperature, stress rupture life under 760 °C/724 MPa was significantly decreased from 125 h to 13 h, while that under 980 °C/200 MPa was not sensitive to the solid solution temperature. Moreover, 1185 °C was selected as the best solution temperature. However, these studies paid more attention to the influence of solid solution temperature, and the effects of the cooling rate after solid solution treatment are rarely mentioned.

Since the precipitate behavior of the secondary γ′ phase is related to the solid solution cooling stage, the size and morphology of γ′ would be affected significantly by the corresponding cooling rate. Pei et al. [20] found that, with a cooling rate of 2 K/s after solution treatment, the primary γ′ precipitate was fine and of a sharp cubic form, while the γ′ phase turned to coarse precipitate after furnace cooling (0.2 K/s). Sajjadi et al. [21] studied the microstructure of UDIMET 500 and found that, with the cooling rate of partial solution decreasing from 186 °C/min to 3.85 °C/min, the weight fraction of γ′ increased from 2% to 26%. Rapid cooling inhibited the precipitation of γ′, and the fine γ′ phases had difficulty growing even during long-term aging. The size and quantity of γ′ significantly impact the mechanical properties and deformation mechanisms of MAR-M247 due to the precipitation strengthening effect [21,22,23,24]. Therefore, the effect of the cooling rate after solid solution treatment on MAR-M247 cannot be ignored [25].

In this work, after the solid solution heat preservation of MAR-M247 alloys, three different cooling methods (water quenching, air cooling, and furnace cooling) were selected to achieve different cooling rates. The effect of cooling rate on microstructure, hardness, and room temperature tensile properties was studied. Moreover, the deformation mechanisms of samples with different cooling rates were compared to explain the variations in mechanical properties. This work is beneficial for further understanding the influence mechanism of the heat treatment process on the mechanical properties of MAR-M247 alloys. In addition, it provides certain guidance for the control of cooling rates in industrial production.

## 2. Materials and Methods

### 2.1. Experimental Materials

MAR-M247 alloy was used as the raw material in this work, the measured chemical compositions of which are shown in Table 1. After investment casting, the samples were heat-treated under non-vacuum conditions. To investigate the effect of cooling rate on microstructure and mechanical properties, the cooling method after solid solution treatment was varied. After solid solution heat treatment at 1185 °C for 2 h, samples were cooled with three different methods: water quenching, air cooling, and furnace cooling. For simplicity, samples are hereafter named “WQ”, “AC”, and “FC”, according to their cooling methods. For the WQ and AC samples, a contactless thermal gun was used to measure their temperature reductions per 2 s or 60 s, respectively. As for FC, we recorded the temperature value displayed on the muffle furnace dashboard every 3 min. The temperature measurement was stopped until the sample temperature was reduced to 500 °C. After calculation, the cooling rates of WQ, AC, and FC were close to 400 °C/s, 1.5 °C/s, and 0.1 °C/s, respectively. All samples were subsequently aged at 870 °C for 20 h, followed by air cooling. After heat treatment, cylindrical specimens with a gauge dimension of φ5 × 25 mm were fabricated for tensile tests.

### 2.2. Mechanical Testing

To investigate the cooling rate-induced hardness change, microhardness was measured using a VMHT30M microhardness tester equipped with a Vickers diamond indenter. The load of 1000 g was applied for 15 s.

The tensile tests were performed in an electronic universal testing machine (LD26-100 kN) at room temperature, according to HB 5143-1996, with an accuracy of ±0.5%. The strain rate was 0.5 mm/min, and two identical samples of each cooling method were tested. Moreover, some interrupted tests were conducted after yielding to identify the deformation mechanism.

### 2.3. Microstructural Characterization

For microstructure observation, the samples were etched chemically in a solution of 33 mL HNO_3_ + 33 mL CH_3_COOH + 33 mL H_2_O + 1 mL HF. Optical microscope (OM, ZEISS Axio Imager A2m, Jena, Germany) and field emission scanning electron microscopy (SEM, ZEISS Supra55) were employed to characterize the morphologies of grain, eutectic, MC carbide, and γ′ phase. After binarization, the size and volume fraction of γ′ were counted by area method via Image Pro Plus 6.0 software.

Dislocation observations were examined by transmission electron microscope (TEM, FEI Tecnai G220, Hillsboro, OR, USA) on the cross sections and by electron channeling contrast imaging technology (ECCI, ZEISS, Germini450) on the longitudinal sections. The cross sections of the interrupted and fractured samples were mechanically ground to a thickness of 70 μm and thinned by double jet electrolysis with an electrolyte of 8% HClO_4_ and CH_3_CH_2_OH. The longitudinal sections of fractured samples were vibration polished with 0.2 μm colloidal silica for two hours after mechanical grinding and polishing.

## 3. Results and Discussion

### 3.1. Effect of Cooling Rate on Microstructure

**Figure 1:**
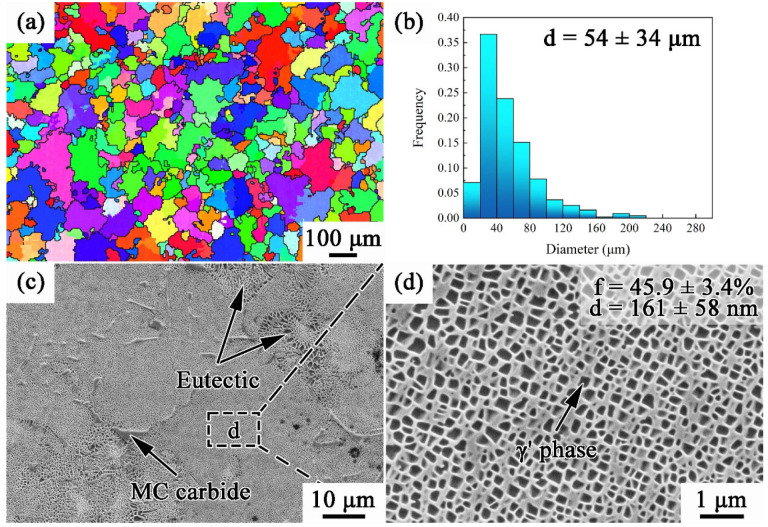
Microstructure of as-cast MAR-M247 alloy: (**a**) grain morphology; (**b**) grain size distribution; and (**c**,**d**) precipitate phase.

**Figure 2:**
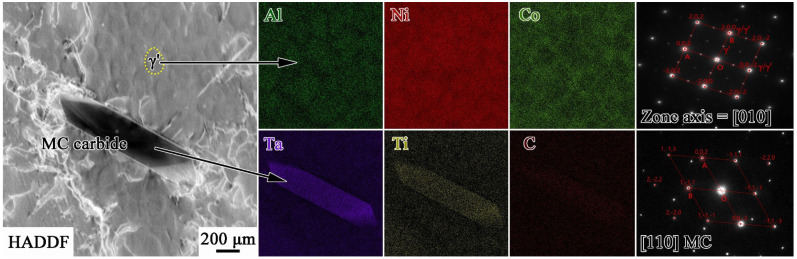
Transmission electron micrograph with corresponding composition profiles and selected area diffraction patterns of γ′ and MC carbide.

**Figure 3:**
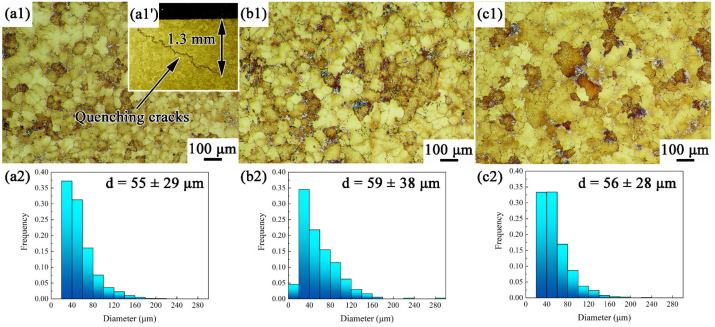
Grain microstructures and size distributions of MAR-M247 alloys with different cooling rates: (**a1**) WQ, grain morphology; (**a1′**) WQ, quenching crack; (**b1**) AC, grain morphology; (**c1**) FC, grain morphology; (**a2**) WQ, grain size distribution; (**b2**) AC, grain size distribution; and (**c2**) FC, grain size distribution.

**Figure 4:**
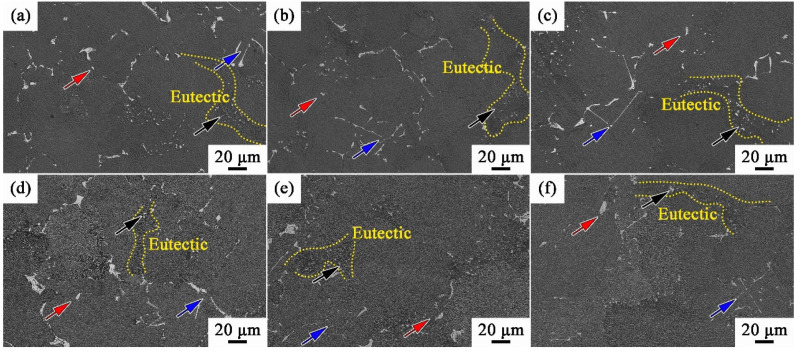
γ/γ′ eutectics and MC carbides of MAR-M247 alloys with different cooling rates: (**a**) WQ, solid solution state; (**b**) AC, solid solution state; (**c**) FC, solid solution state; (**d**) WQ, aging state; (**e**) AC, aging state; and (**f**) FC, aging state. Blue, red and black arrows refer to script-like, block-like, and point-like MC carbides, respectively.

**Figure 5:**
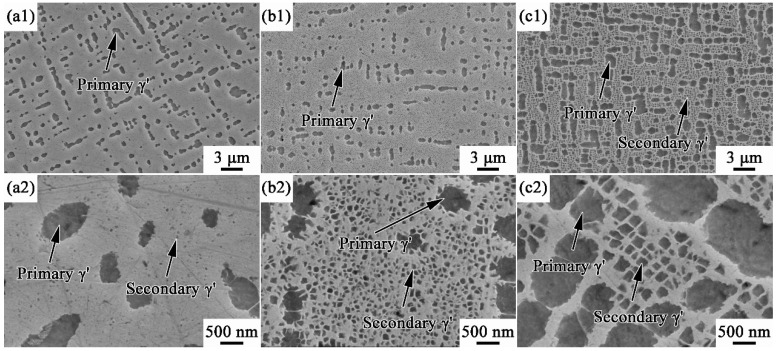
γ′ distributions and morphologies of MAR-M247 alloys after solution treatment: (**a1**) WQ, γ′ distribution; (**b1**) AC, γ′ distribution; (**c1**) FC, γ′ distribution; (**a2**) WQ, γ′ morphology; (**b2**) AC, γ′ morphology; and (**c2**) FC, γ′ morphology.

**Figure 6:**
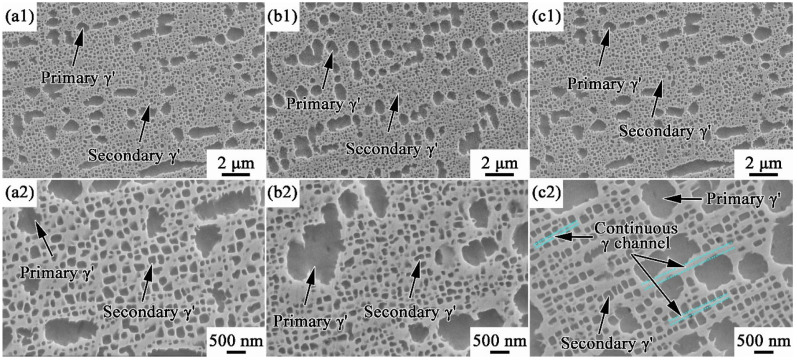
γ′ distributions and morphologies of MAR-M247 alloys after aging treatment: (**a1**) WQ, γ′ distribution; (**b1**) AC, γ′ distribution; (**c1**) FC, γ′ distribution; (**a2**) WQ, γ′ morphology; (**b2**) AC, γ′ morphology; and (**c2**) FC, γ′ morphology.

The microstructures of MAR-M247 alloys are shown in Figure 1, Figure 2, Figure 3, Figure 4, Figure 5 and Figure 6, including γ matrix, γ′ precipitate, γ/γ′ eutectic, and MC carbide.

Figure 1 shows the microstructure of the as-cast MAR-M247 alloy. Grains with an average diameter of 54 μm present equiaxed shape, and there are γ′ phases, MC carbides, and rose-like γ/γ′ eutectics distributed inside grains. It can be seen from Figure 1d that γ′ phases are homogenous and of cubical shapes. The volume fraction of γ′ in the as-cast alloy is 45.9%, and the average size of γ′ is 161 nm. The precipitates mentioned above have been identified by TEM. The corresponding composition profiles and selected area electron diffraction (SAED) patterns are shown in Figure 2. The γ′ phases are rich in Al, Ti, and are coherent with the γ matrix, while the MC carbides are Ta/Ti-rich.

Figure 3 presents the grain microstructures and size distributions of MAR-M247 alloys with different cooling rates after heat treatment. The average grain size of WQ, AC, and FC are in the range of 55~59 μm, suggesting that the grain size is not affected by the cooling rate after solid solution treatment in this work. Therefore, the effect of grain size on the varied tensile behaviors could be excluded in the subsequent discussion. In addition, aggressive cooling would cause severe distortion and even quench cracking due to the excessive thermal stress [26,27,28]. As shown in Figure 3a1′, an intergranular quenching crack initiates from the sample surface during the water quenching process. The crack depth is up to 1.3 mm, approximately 20% of the gauge diameter of the tensile sample. Quenching cracks not only reduces the actual bearing area but also causes localized stress concentration. This would lead to accelerated failure and seriously deteriorate the tensile properties.

Figure 4 shows the distributions and morphologies of γ/γ′ eutectics and MC carbides of MAR-M247 alloys after solid solution treatment (Figure 4a–c) and aging treatment (Figure 4d–f). The γ/γ′ eutectics are distributed along the grain boundaries, as cycled by the yellow dashed lines in Figure 4. There are three types of MC carbides that can be observed: script-like carbides (marked by blue arrows), block-like carbides (marked by red arrows), and point-like ones (marked by black arrows). The script-like and block-like carbides are distributed randomly, while the point-like ones are mainly distributed in γ/γ′ eutectics. It can be seen that the distributions of point-like carbides and γ/γ′ eutectics are overlapping in Figure 4. The characteristics of γ/γ′ eutectics and MC carbides are almost the same in WQ, AC, and FC, indicating that the cooling rate after solid solution has few influences on eutectics and MC carbides. Moreover, by comparing Figure 4a–f, it is found that the eutectics and MC carbides are not affected by the aging treatment. This is because the heat treatment temperatures (1185 °C and 870 °C) are much lower than the dissolution temperature of eutectics and MC carbides, which are not high enough for the dissolution of eutectics and MC carbides. Therefore, the effect of eutectics and MC carbides on the varied tensile behaviors could be excluded in the subsequent discussion.

Figure 5 presents the distributions and morphologies of γ′ after solid solution treatment with different cooling rates. There are two types of γ′ that can be observed. Since a sub-solid solution temperature of 1185 °C was selected in this work, there are some incompletely dissolved γ′ phases remaining after solid solution treatment, which are named primary γ′ here. In addition, some nano-sized secondary γ′ phases precipitate during the cooling stage after solid solution treatment. It can be observed in Figure 5 that the cooling rate significantly affects the morphology, volume fraction, and size of γ′ phase, especially the secondary γ′. As shown in Figure 5 and Table 2, the volume fraction and size of primary γ′ in WQ are 13.8% and 543 nm. With the cooling rate decreasing from 400 °C/s to 1.5 °C/s, the volume fraction (11.1%) and size (547 nm) of primary γ′ in AC have almost no changes compared to those in WQ. When the cooling rate further slows down to 0.1 °C/s, significant coarsening of primary γ′ occurs, and the corresponding volume fraction and size increase to 35.7% and 865 nm, respectively. In addition, the primary γ′ phases in WQ and AC present spherical shapes (Figure 5a2,b2), while those in FC are close to cubical shapes.

As the cooling rate decreases, the coarsening of secondary γ′ occurs, and the volume fraction of secondary γ′ also decreases. Moreover, the morphology of secondary γ′ transitions from spherical shape to cubical shape. Since the cooling rate of WQ is 400 °C/s, there is not enough time for Ti/Al to diffuse from the surrounding γ matrix to secondary γ′, and the growth of secondary γ′ is inhibited. As a result, the secondary γ′ phases are too small and show a point-like shape in Figure 5a2, which is difficult for quantitative statistics. Owing to its medium cooling rate (1.5 °C/s), secondary γ′ phases in AC exhibit a medium size of 76 nm with a volume fraction of 25.9%, and both spherical and cubical phases can be observed in Figure 5b2. Treated by the lowest cooling rate (0.1 °C/s), the secondary γ′ phases in FC present coarse and cubical shapes (Figure 5c2), and their average size and volume fraction are 190 nm and 20.7%, respectively.

The shape change is related to the growth of γ′ phase. As known, there is a competition between the interface energy mechanism and the strain energy mechanism during the growth of γ′ phase [29,30,31]. Fast cooling suppresses the growth of secondary γ′. As a result, the dominant interface energy mechanism forces the γ′ to be spherical to minimize the total interface energy [30]. When the cooling slows down, the γ′ phases of FC have enough time to grow. The strain energy mechanism dominates at this time, and the γ′ phases exhibit a cubical shape to reduce the total strain energy [31].

Figure 6 presents the distributions and morphologies of γ′ after aging treatment. The primary γ′ phases remain spherical shapes but become more regular (Figure 6a1–c1), while the secondary γ′ phases are close to cubical shapes (Figure 6a2–c2). Coarsening of both primary and secondary γ′ phases occurs in all samples, and the corresponding statistical results are presented in Table 2. The total volume fractions of γ′ phases are almost the same in all samples (45~49%), but the proportion of primary γ′ increases as the cooling rate decreases. In addition, the sizes of primary γ′ (775 nm) and secondary γ′ (113 nm) in WQ are close to those in AC (795 nm and 115 nm), while the FC ones are significantly larger, reaching 936 nm and 156 nm, respectively. Moreover, the distribution of γ′ phases in WQ and AC are relatively disordered, where the matrix channels are separated by γ′. On the contrary, continuous perpendicular γ channels form in FC, which are longer and wider than those in WQ and AC. The continuous wide γ channels would provide larger mean free paths for dislocations to slip, which is beneficial for the deformation uniformity.

Comparing Figure 5 and Figure 6, it can be found that γ′ phases in all samples have grown up during aging treatment except the secondary γ′ in FC. The coarsening of γ′ depends on the diffusion of Al and Ti from the surroundings. Once Al and Ti in the adjacent matrix are consumed, farther solute atoms, including those in smaller γ′, will be absorbed to continue the coarsening [32,33]. As a result, the volume fraction and size of secondary γ′ in FC decrease from 20.7%, 190 nm to 14.6%, 156 nm, while significant coarsening occurs in primary γ′.

From the above results, it is found that there is no significant difference between the microstructures of WQ and AC, whether in a solid solution state or aging state. This indicates that the microstructure of MAR-M247 alloy is not sensitive to the solid solution cooling rate between 1.5 °C/s and 400 °C/s. On the contrary, microstructures, mainly γ′ phases, are significantly affected by the cooling rate range of 0.1~1.5 °C/s. Hence, more attention should be paid to the solid solution cooling rate control in the actual production, especially in the range of 0.1~1.5 °C/s.

### 3.2. Effect of Cooling Rate on Mechanical Property

**Figure 7:**
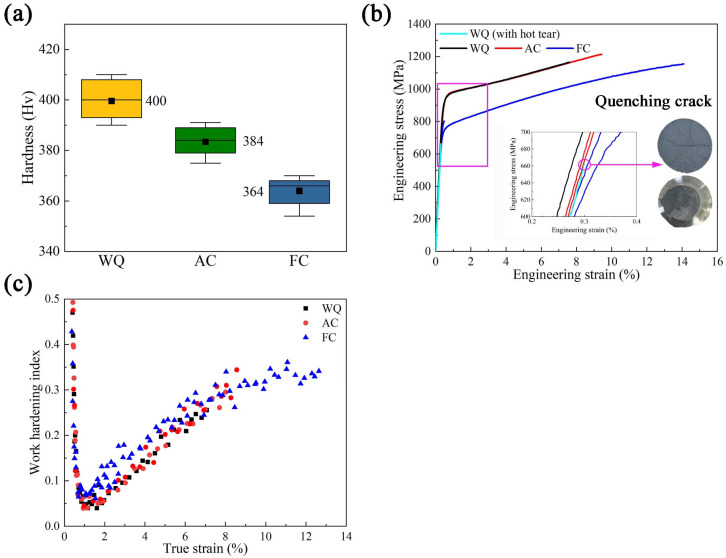
Mechanical properties of MAR-M247 alloys with different cooling rates: (**a**) hardness; (**b**) engineering stress–strain curve; and (**c**) work hardening index.

Figure 7 shows the mechanical properties of MAR-M247 alloys with different cooling rates. As shown in Figure 7a, WQ has the highest hardness of 400 HV. With a further decrease in cooling rate, the hardnesses of AC and FC decrease to 384 HV and 364 HV, respectively. Figure 7b presents the engineering stress–strain curves, and the corresponding mechanical parameters are listed in Table 3.

As illustrated in Figure 7b, premature fracture accidentally occurs outside the gauge section during the tensile test of WQ due to the quenching cracks. Considering this, the strengths and elongation of WQ are not listed in Table 3 to avoid misleading, but the corresponding stress–strain curves are retained to analyze work-hardening behavior. All curves show the same tendency that there is continuous work-hardening after yielding. The ultimate tensile strength (UTS), yield strength (YS), and elongation (EL) of AC are 1210 MPa, 960 MPa, and 8.5%, respectively. When the cooling rate slows down to 0.1 °C/s, the UTS and YS decrease to 1167 MPa and 771 MPa, while the EL increases to 13.5%.

Combined with the hardness and tensile properties, it can be concluded that a decrease in cooling rate will deteriorate the room temperature strengths of the MAR-M247 alloy but increase its elongation significantly, reflecting the inherent conflict between strength and plasticity. Except for the γ′ phase, other microstructure characterizations, including grain size, γ/γ′ eutectic, and MC carbide, are almost not affected by the cooling rate. Therefore, the decreased yield strengths in this work are attributed to the coarsening of γ′ phases since there are no significant differences between the total γ′ volume fractions of WQ, AC, and FC. According to Galindo-Nava et al. [34,35], there is a critical diameter of γ′ that would contribute to the highest precipitation strengthen effect. As a certain volume fraction, γ′ with a closer size to the critical diameter provides a higher strengthening effect. For MAR-M247 alloy, the critical diameter is calculated to be 74 nm [36], smaller than those of γ′ phases in all aging state samples. Therefore, with the coarsening of γ′ phases, the effect of precipitation strengthening is gradually weakened. As a result, the yield strength of FC is decreased by 189 MPa compared to that of AC. In addition, the hardnesses, as well as the ultimate tensile strengths, of WQ, AC, and FC decrease in turn as their γ′ phases become larger, resulting from the slowed cooling rate.

Figure 7c presents the work-hardening curves of WQ, AC, and FC. All samples undergo work-hardening periods after yielding, and FC always has a larger work-hardening index than WQ and AC. Moreover, the elongation of FC can reach 13.5%, which is significantly higher than the 8.5% of AC and represents a 59% increase. The elongation is determined not only by the work-hardening level but also by the sustained time of work-hardening [37]. Both of the above aspects are affected by the deformation mechanism, which will be discussed in Section 3.3 to investigate the origins of the excellent elongation of FC.

### 3.3. Effect of Cooling Rate on Deformation Mechanism

**Figure 8:**
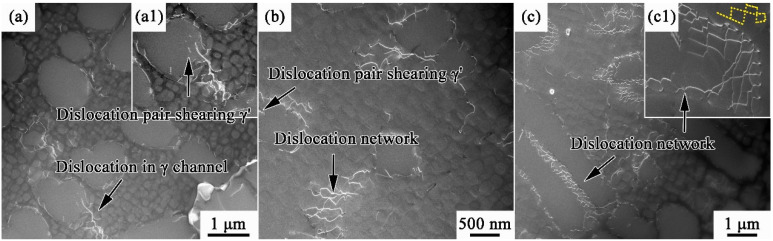
Dislocation configurations of MAR-M247 after yielding: (**a**) WQ; (**b**) AC; and (**c**) FC. (**a1**) and (**c1**) are local amplifications of dislocation configurations to present dislocation shearing and dislocation network.

The dislocation configurations of WQ, AC, and FC after yielding are shown in Figure 8. At this stage, the dislocation densities in all samples are relatively low since the plastic deformation has just begun. Dislocations initiate from the γ matrix, and their glide motions are hindered by γ′ precipitates. As a result, dislocations pile up at the γ/γ′ interfaces and tangle with each other. The dislocation accumulation causes local stress concentration, forcing dislocations to shear γ′ in the form of dislocation pairs, as shown in Figure 8a1,b. In addition, a few irregular dislocation networks are observed in AC (Figure 8b), while massive dislocation networks exist in FC in regular square shapes (Figure 8c1).

**Figure 9:**
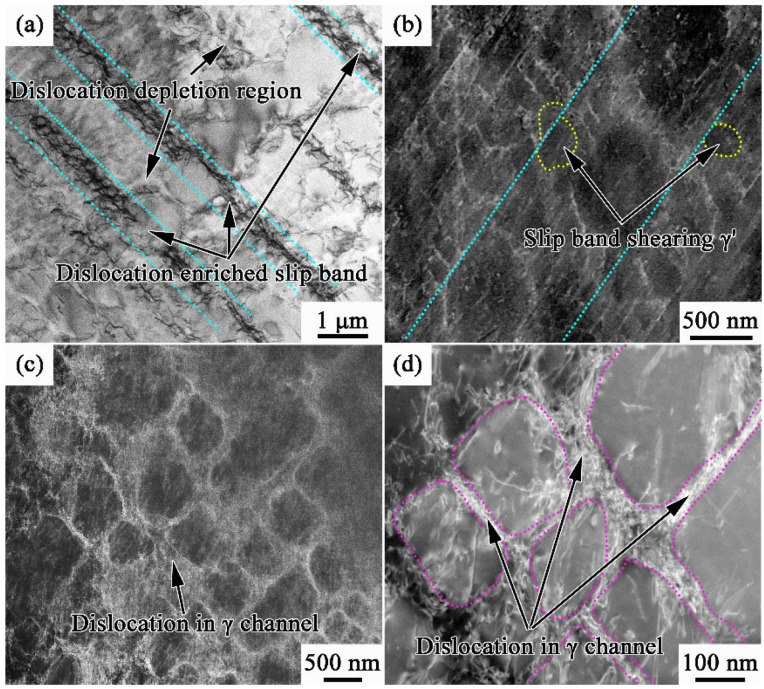
Dislocation configurations of fractured MAR-M247: (**a**,**b**) AC; (**c**,**d**) FC.

Figure 9 presents the deformation structures of AC and FC after fracture. Considering that WQ has premature fracture outside the gauge section due to the quenching cracks, its deformed structure is not compared with AC and FC here to avoid misleading. Compared with those at the yielding state, the dislocation densities in fractured samples increase significantly. Moreover, the dislocation density of FC is much higher than that of AC. This is related to the higher elongation of FC since FC has suffered more deformation before fracture. An interesting fact is that the deformation structures of AC and FC are completely different. As shown in Figure 9a, there are massive slip bands in AC, and the dislocations are mainly concentrated inside slip bands. Moreover, there are few dislocations between the slip bands, forming several dislocation depletion zones. Figure 9b is an enlarged image of the deformation structures in AC, and it can be observed that γ′ phases are sheared by slip bands (marked by the yellow dotted lines). The dislocation distribution in FC is more uniform, as shown in Figure 9c. In addition, no-slip bands are observed in all TEM fields. After local amplification (Figure 9d), it can be found that dislocations are concentrated in the γ matrix. The γ′ phases are basically not sheared by dislocations, keeping cubical shapes.

The dislocation networks result from the reaction of two sets of dislocations in the matrix channels [38,39]. When the dislocation motion is hindered by the precipitates, it will bow out to the neighboring γ matrix and cross-slip to another slip plane. Once these dislocations move to the same slip plane and come across each other, the networks may be formed by the reaction of dislocations. Therefore, the formation of a dislocation network is related to the difficulty of dislocation bowing out, which is affected by the width of γ channel [40]. The γ matrix channels of FC are wider than those of WQ and AC, which are beneficial for dislocations bowing out to form dislocation networks. For AC, massive slip bands shear γ′ phases, weakening the precipitation strengthening effect. At the same time, the dislocation networks in FC could prevent γ′ from being sheared and would further hinder the movement of subsequent dislocations. Therefore, the work-hardening index of FC is higher than that of AC, as shown in Figure 7c. In addition, the dislocation accumulation in slip bands would lead to severe plastic localization in AC. The deformations of slip bands and the dislocation depletion zones are difficult to coordinate. As a result, cracks would easily initiate from their interfaces and lead to fractures soon. On the contrary, the relatively uniform dislocation distribution in FC homogenizes the plastic strain distribution and, therefore, delays the occurrence of uneven deformation, leading to the improved elongation of FC.

## 4. Conclusions

In this work, experiments in cooling from a subsolvus temperature of 1185 °C with different cooling methods (water quenching, air cooling, furnace cooling) were conducted. After the same aging treatment, microstructure characterizations and mechanical tests were carried out on all samples. The effect of the cooling rate on the microstructure and mechanical properties of MAR-M247 alloys was analyzed. The following conclusions can be drawn:

1. The cooling rate after solid solution treatment mainly affects the precipitation behavior of the secondary γ′. Except for γ′ precipitates, other microstructure characterizations of MAR-M247 alloy, including grain size, eutectic, and MC carbide, are not sensitive to the change in the cooling rate after solid solution treatment.
2. The cooling rate significantly affects the size and morphology of γ′ phases, especially the secondary γ′, while it has few effects on the total volume fraction of γ′ phase. As the cooling rate decreases, γ′ phases are coarser and more cubical.
3. Decreasing the cooling rate significantly deteriorates the hardness and room-temperature strengths of MAR-M247 alloy, while it is beneficial for the improvement of elongation.
4. As the cooling rate slows down, the tensile deformation mechanism is transformed from dislocation shearing to Orowan bypassing, leading to a weaker precipitation strengthening effect and lower tensile strength. The resulting interface dislocation networks significantly improve the work-hardening index and deformation uniformity, resulting in higher elongation.
5. The microstructure and mechanical properties of MAR-M247 alloys are sensitive to the change in the cooling rate between 0.1 °C/s and 1.5 °C/s after solid solution treatment, which needs special attention in industrial production. The changed cooling rate between 1.5 °C/s and 400 °C/s has few effects on the microstructure and room-temperature mechanical properties of MAR-M247 alloys.

## Figures and Tables

**Table 1:** Chemical composition of the MAR-M247 alloy (wt.%).

C	Cr	Co	W	Mo	Ta	Al	Ti	Hf	B	Zr	Ni
0.16	8.7	10.6	10.3	0.63	3.0	5.4	1.05	1.5	0.012	0.053	Bal.

**Table 2:** Volume fraction and size of γ′ precipitate treated by different cooling rates after solid solution.

	After Solution Treatment				After Aging Treatment			
	Volume Fraction (%)		Size (nm)		Volume Fraction (%)		Size (nm)	
	Primary γ′	Secondary γ′	Primary γ′	Secondary γ′	Primary γ′	Secondary γ′	Primary γ′	Secondary γ′
WQ	13.8 ± 2.1	--	543 ± 21	--	21.1 ± 1.7	23.9 ± 3.3	775 ± 19	113 ± 10
AC	11.1 ± 2.3	25.9 ± 1.9	547 ± 13	76 ± 12	25.7 ± 0.6	22.6 ± 2.5	795 ± 17	115 ± 9
FC	35.7 ± 3.7	20.7 ± 4.6	865 ± 15	190 ± 21	34.4 ± 2.4	14.6 ± 1.8	936 ± 36	156 ± 16

-- The secondary γ′ phases in solution treated WQ were too small to be statistically analyzed.

**Table 3:** Hardness and room temperature tensile property of MAR-M247.

	Hardness (HV)	UTS (MPa)	YS (MPa)	EL (%)	E (GPa)
WQ	400 ± 8	--	--	--	205
AC	384 ± 6	1210 ± 13	960 ± 11	8.5 ± 0.6	205
FC	364 ± 6	1167 ± 9	771 ± 1	13.5 ± 0.2	205

-- The tensile property parameters of WQ were not obtained because fracture occurred outside the gauge section.

## Data Availability

Data are available from the authors upon reasonable request.
